# Supplementary material for: Horned Melon Pulp, Peel, and Seed: New Insight into Phytochemical and Biological Properties
Source: Antioxidants (Basel). 2022 Apr 23;11(5):825. doi: 10.3390/antiox11050825 (PMC9137901; doi:10.3390/antiox11050825)
Supplement: Supplementary file 1 [file antioxidants-11-00825-s001.zip › antioxidants-1686390-supplementary/Supplement Table S1.pdf]

**Supplement Table S1.** The effect of controls during disk-diffusion method.

| Test organism | | Inhibition zone (mm) (mean ± stand. dev.) | | |
|---|---|---|---|---|
| | | Positive controls (antibiotic/antimycotic) | | Negative control |
| | | Clavulanic acid | Actidione | Water |
| Gram (+) bacteria | *B. cereus* | 24.0±0.0 | - | nd* |
| | *S. aureus* | 28.3±0.6 | - | nd |
| | *E. faecalis* | 28.0±1.0 | - | nd |
| Gram(-) bacteria | *E. coli* | 27.0±0.0 | - | nd |
| | *P. aeruginosa* | 21.0±0.0 | - | nd |
| | *S. Typhimurium* | 29.3±0.6 | - | nd |
| Yeasts and fungi | *S. cerevisiae* | - | 34.0±0.0 | nd |
| | *C. albicans* | - | 37.0±0.0 | nd |
| | *A. brasiliensis* | - | 27.0±0.0 | nd |
| | *P. aurantiogriseum* | - | 28.0±0.0 | nd |

* nd – not detected
