# Supplementary figures and images for: Horned Melon Pulp, Peel, and Seed: New Insight into Phytochemical and Biological Properties
Source: Antioxidants (Basel). 2022 Apr 23;11(5):825. doi: 10.3390/antiox11050825 (PMC9137901; doi:10.3390/antiox11050825)

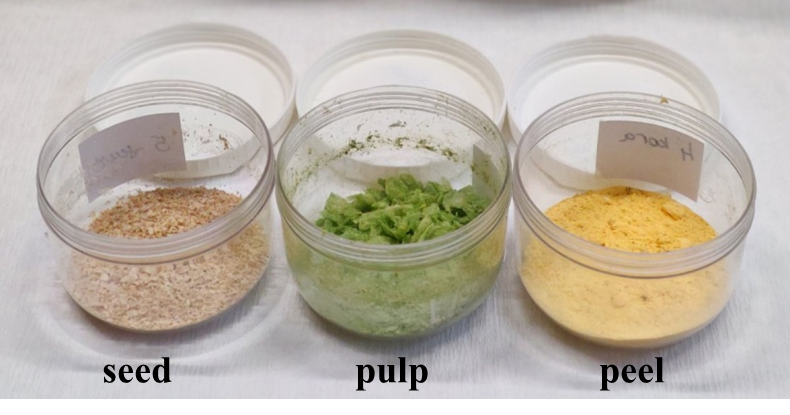

Supplement: Supplementary file 1 [file antioxidants-11-00825-s001.zip › antioxidants-1686390-supplementary/Supplement Figure S1 The prepared samples for extraction.png]
